# Cabbage Leaf Epicuticular Wax Deters Female Oviposition and Larval Feeding of *Pieris rapae*

**DOI:** 10.1007/s10886-025-01597-z

**Published:** 2025-03-25

**Authors:** Itsuki Ueno, Taisei Kanedawara, Kodai Inoue, Sotaro Watanabe, Hisashi Ômura

**Affiliations:** 1 Graduate School of Integrated Sciences for Life, Hiroshima University, Higashihiroshima, 739-8528 Japan; 2 School of Applied Biological Science, Hiroshima University, Higashihiroshima, 739-8528 Japan; 3 Seto Inland Sea Carbon-Neutral Research Center, Hiroshima University, Higashihiroshima, 739-8528 Japan

**Keywords:** Small white butterfly, Cabbage leaf, Epicuticular wax, Long-chain alkane, Host suitability, Pest resistance

## Abstract

The small white butterfly *Pieris rapae* is a major pest of brassicaceous crops, causing extensive damage to cabbage *Brassica oleraceae* var. *capitata*. *Pieris rapae* utilizes glucosinolates, which are characteristic of brassicaceous plants, for host selection. However, the effect of epicuticular wax of cabbage leaves on host selection by *P*. *rapae* remains unclear. This study was aimed to determine the effects of leaf epicuticular wax on female oviposition and larval feeding of *P*. *rapae*. We used a common cabbage variety ‘Kinkei 201’ containing *n*-nonacosane as the predominant leaf wax component. Wax removal treatments, in which the leaf surface was rubbed with cotton balls, significantly increased female oviposition and mid-stage (2nd–4th instars) larval feeding compared with untreated (intact) leaves. Spraying treatments with *n*-nonacosane standard on wax-removed leaves significantly suppressed female oviposition and mid-stage larval feeding. Thus, *n*-nonacosane in cabbage leaf epicuticular wax potentially affects host selection and preference by *P*. *rapae*. In contrast, final-stage (5th instar) larvae significantly preferred intact leaves to wax-removed leaves, and spraying treatments with *n*-nonacosane did not significantly change their feeding on wax-removed leaves. This suggests that young larvae are more susceptible to wax feeding inhibition than older larvae and that increasing leaf wax content enhances the resistance of cabbage against *P. rapae*. Acquiring these traits in cabbage leaves through improved breeding and cultivation methods may reduce and control the oviposition and feeding damage by *P*. *rapae*.

**Supplementary Information:**

The online version contains supplementary material available at 10.1007/s10886-025-01597-z.

## Introduction

*Brassica oleracea* L. (Brassicaceae) is an economically important crop that includes cabbage, broccoli, cauliflower, Brussels sprouts, collards, kale, and kohlrabi (Stoner 1990; Zandberg et al. 2022). Management of *Brassica* crop pests relies primarily on the application of chemical pesticides, which has led to the development of insecticide resistance (Obermeier et al. 2022; Yu and Nguyen 1992). Therefore, attempts have been made to evaluate naturally occurring pest resistance in *B*. *oleracea* and to breed resistant lines and cultivars (Liu et al. 2021; Mitchell et al. 2016).

Several lines and cultivars of *B*. *oleracea* exhibit high resistance to certain pest species (Pimentel 1961; Vail et al. 1991). For example, a cauliflower (*B*. *oleracea* var. *botrytis*) line from Australia and several cabbage (*B*. *oleracea* var. *capitata*) lines developed at the New York Agricultural Experimental Station have considerably high resistance to lepidopterous pests including the small white butterfly *Pieris rapae* (Lepidoptera: Pieridae), the cabbage looper *Trichoplusia ni* (Lepidoptera: Noctuidae), and the diamondback moth *Plutella xylostella* (Lepidoptera: Plutellidae) (Dickson and Eckenrode 1975, 1980; Dickson et al. 1984, 1986; Eckenrode et al. 1986; Edelson and Dickson 1988; Shelton et al. 1988; Stoner 1990). These lines are characterized by dark green and glossy leaves with low-wax blooms, suggesting that leaf wax traits are responsible for resistance.

The aboveground surfaces of terrestrial higher plants have a bilayer matrix called the cuticle (Eglinton and Hamilton 1967; Müller and Riederer 2005). The inner layer, called the cuticular layer, is composed of the polyester polymer cutin, whereas the outer layer, called the cuticular proper, comprises cutin and wax (intracuticular wax). The outside of the cuticle proper is covered with lipophilic wax (epicuticular wax). The epicuticular wax is a mixture of long-chain aliphatic compounds, which in *B*. *oleracea* are mostly *n*-alkanes, aldehydes, primary and secondary alcohols, and ketones (Laila et al. 2017; Macey and Barber 1970). The epicuticular wax forms amorphous or crystalline layers, giving rise to a surface coating known as wax bloom (Baker 1974; Eglinton and Hamilton 1967). The primary role of epicuticular wax is to prevent water loss, but its species-specific chemical composition and variable crystal microstructure are involved in mediating trophic interactions between plants and herbivores (Eigenbrode and Espelie 1995; Müller and Riederer 2005). In general, the plant phenotypes called ‘glossy’ have relatively simple microstructures, different compositions, and smaller amounts of epicuticular wax than the normal phenotypes called ‘waxy’ (Eigenbrode and Espelie 1995).

Notably, the glossy lines of *B*. *oleracea* are not necessarily more pest resistant than the waxy lines, and their resistance varies with pest species, plant growth stages, and experimental conditions (Eckenrode et al. 1986; Shelton et al. 1988; Stoner 1990, 1992). For example, the glossy lines harbor lower populations of cabbage aphid *Brevicoryne brassicae* (Hemiptera: Aphididae) and are less infested by *P*. *xylostella* larvae than the waxy lines, but receive more feeding damage by flea beetles *Phyllotreta cruciferae* (Coleoptera: Chrysomelidae) and *Phyllotreta striolata* (Coleoptera: Chrysomelidae) and more egg-laying by* P*. *xylostella* adults (Stoner 1990; Uematsu and Sakanoshita 1989; Badenes-Pérez et al. 2004). The glossy and waxy lines differ not only in leaf epicuticular wax compositions but also in various traits such as leaf color, trichomes, and glucosinolates, each of which may be involved in pest resistance (Cartea et al. 2010; Dickson et al. 1986; Stoner 1992). Therefore, to directly assess pest resistance due to leaf wax, examining the behavioral responses and preferences of herbivores for different wax profiles on the same plant is necessary (Müller and Hilker 2001; Silva et al. 2017).

*Pieris rapae* is a specialist herbivore of brassicaceous plants and causes extensive damage to broccoli and cabbage. Adult females and larvae of *P. rapae* have distinct selection among different *Brassica* varieties, and various plant components are involved in their host preference (Chew and Renwick 1995; Jõgar et al. 2009). Glucosinolates, particularly those containing an indoyl moiety, play a central role in inducing female oviposition and larval feeding (Hopkins et al. 2009; Miles et al. 2005; Städler and Reifenrath 2009; Badenes-Pérez 2023). Cabbage leaf odors, composed of several general plant volatiles, serve as olfactory cues for host location by females (Ikeura et al. 2010; Itoh et al. 2018). Furthermore, wax components of *Brassica* leaves may affect host selection and preference in *P*. *rapae*. Field observations revealed that glossy broccoli lines had lower populations of *P*. *rapae* larvae than normal lines (Stoner 1990, 1992). Under laboratory observations, a larger portion of larvae moved longer distances on glossy collard leaves than on normal ones, resulting in reduced feeding (Stoner 1997). These results suggest that larvae prefer to feed on the leaves of brassicaceous crops with higher wax contents. However, it has never been investigated which leaf wax components enhance larval feeding. Moreover, to the best of our knowledge, reliable evidence that adult females utilize leaf wax for oviposition is lacking.

For comprehensive understanding of host selection mechanisms of *P*. *rapae* and pest resistance of *Brassica* crops, the effects of leaf epicuticular wax on female oviposition and larval feeding need to be determined. As a first step, the aim of this study was to examine the oviposition and feeding preferences of *P*. *rapae* among leaf surfaces with different wax profiles using a common cabbage variety.

## Materials and Methods

### Plants

A common cabbage cultivar ‘Kinkei 201’ with waxy leaves was used as the plant material for chemical analyses and bioassays. Cabbage seeds were commercially purchased from Sakata Seed Co. (Yokohama, Japan). Planting soil was prepared by mixing potting and sandy soils, both of which were commercially purchased, at a volume ratio of 1:1; sieved using an 8.6 mesh size (2 mm) sieve; and air-dried. Two cabbage seeds were sown in each 1 L plastic pot (12 cm upper outer diameter, 14 cm height) containing approximately 600 g planting soil and maintained under controlled conditions of temperature 20 ± 1 °C and photoperiod 16L:8D. The potted seedlings were irrigated to maintain soil moisture content at 60–80%. Two weeks after sowing, the seedlings were thinned to one seedling per pot. From the fifth weeks after sowing, the irrigation was adjusted to maintain 60% soil moisture content at 60%.

### Insects

*Pieris rapae* individuals were obtained from a laboratory-maintained population derived from wild females captured in Higashihiroshima, Japan. Larvae were reared on fresh leaves of common cabbage cultivars other than Kinkei 201 (e.g., Irodori and Nakawase No. 2) under conditions of temperature 25 °C and photoperiod 16L:8D. Adults were fed 15% sucrose solution daily and maintained individually in transparent plastic cylindrical cups (9 × 12 cm ID). Adult males and females were transferred to a mosquito net cage (105 × 75 × 90 cm) in a greenhouse between 10:00 and 14:00 h on sunny days to allow mating. Females mated on average 2.8 days after emergence. Mated females were collected, reared in the same manner as described above, and used for oviposition bioassays. After the bioassays, mated females were allowed to lay eggs freely on potted cabbages, and the eggs and hatched larvae were used for feeding bioassays and population maintenance.

### Cabbage Leaf Wax Extraction

Fourth leaves from the top of 7-week-old seedlings of cabbage cv. Kinkei 201 were used for chemical analyses of leaf wax. One leaf from each seedling was detached at the petiole base and soaked in 50 mL chloroform for 30 s to extract epicuticular wax. Thereafter, each leaf was placed between transparent glass plates and photographed using a TG-6 digital camera (Olympus, Tokyo, Japan) and the leaf area was determined using ImageJ 1.54d image analysis software. The sampling was repeated ten times with different seedlings and each leaf extract was stored separately at −20 °C until use.

### Chemical Analysis

The cabbage leaf wax extracts were subjected to gas chromatography-electron impact mass spectrometry (GC-EIMS). Prior to analyses, a 2 mL portion of each sample was concentrated to 200 μL under a gentle nitrogen stream at 60 °C. GC-EIMS was carried out at an EI voltage of 70 eV using a QP5000 mass spectrometer (Shimadzu, Kyoto, Japan) and GC-17A gas chromatograph (Shimadzu, Kyoto, Japan) equipped with a Supelco Equity-1 capillary column (15 m × 0.25 mm ID, 0.25 μm film thickness: Bellefonte, PA, USA). The splitless injection of 1 μL concentrated sample was performed with an injector temperature of 280 °C and a split opening 30 s after injection. The oven temperature was programmed from 50 °C (initial 2 min hold) to 280 °C (final 10 min hold) at 10 °C/min. The major components detected were identified by comparing their retention times and mass spectra with those of authentic chemicals or were tentatively identified based on published data. *n*-Nonacosane (nC29) in each sample was quantified with a calibration curve of its authentic chemical (Online Resource 1). Other components were semi-quantified by comparing their peak areas with that of nC29. Then, the content of each component per leaf area was calculated.

### Leaf surface Treatments

Cabbage cv. Kinkei 201 leaves used for bioassays (average area 38.2 cm^2^) were detached from the petiole base of over 7-week-old seedlings. The following two treatments were applied to modify the leaf wax properties.

1. Mechanical removal of epicuticular wax: the innate epicuticular wax was removed by rubbing both the adaxial and abaxial sides of the leaf with clean cotton balls, avoiding damage to the surface, thus changing the leaf surface from glaucous to glossy. The remaining wax on the treated leaf surface was extracted using the method described above and analyzed using GC–MS, and the most predominant component was reduced to 37% of its original content in the untreated leaves (Online Resource 2).
2. Spraying with an authentic compound: authentic nC29 was sprayed on the leaf surface, immediately after mechanical removal of epicuticular wax, using a commercial hobby airbrush (0.3 mm nozzle size, 25 psi maximum air pressure). Certain amounts of nC29 (0.1, 1, 5, and 10 mg) were dissolved in 7 mL isopentane, filled into an airbrush, and sprayed onto both the adaxial and abaxial leaf sides from a distance of 15 cm. Leaves sprayed with nC29 were used as treatments, and leaves sprayed with 7 mL solvent only in the same manner were used as controls. When spraying onto either the right or left half from the midvein of a single leaf, one half of the leaf was covered with aluminum foil to prevent the compound from being applied. After spraying, the leaves were air-dried until the solvent was completely evaporated and then used for bioassays. When 0.1, 1, and 10 mg nC29 was sprayed on both sides of half leaf, the compound content on the leaf surface was 7, 56, and 360 µg/cm^2^, respectively (Online Resource 3).

### Leaf Surface Morphology

Scanning electron microscopy (SEM) was performed to examine the surface microstructure of the cabbage leaves used in the bioassays. Glaucous leaves detached from seedlings were used intact or after implementation of either of the surface treatments described above: (1) mechanical removal of epicuticular wax or (2) mechanical wax removal, followed by spraying with an isopentane solution containing 5 mg nC29. A test piece (5 × 5 mm) was cut from each leaf sample and attached to an SEM cylinder mount using double-sided tape. The leaf on the mount was rapidly frozen using an FDC10 cooling unit (SUN Technologies, Tokyo, Japan) at −100 °C, dehydrated using an FD-6510 freeze dryer (SUN Technologies, Tokyo, Japan), and then sputter-coated with Pt using a JFC-1600 auto fine coater (JEOL, Akishima, Japan). The leaf surface morphology was examined using a JSM-5610 LV SEM (JEOL, Akishima, Japan) at 15 kV.

### Oviposition Bioassay

The effect of leaf wax on female oviposition preference was examined using two-choice tests in which one female was allowed to lay eggs freely on one cabbage leaf (average area: 26.7 cm^2^) with different wax traits on the left and right halves. The left or right half of each test leaf was randomly treated with one of the following combinations of leaf surface treatments: (1) mechanical wax removal vs. no treatment (leaf wax intact); (2) wax removal, followed by spraying with solvent vs. wax removal, followed by spraying with nC29 (0.1, 1, and 10 mg) isopentane solution. After air-drying, each test leaf was placed vertically in a 5 mL glass vial containing water and placed at the center of a plastic test chamber (33 × 42 × 24 cm) equipped with a transparent lid.

The bioassays were conducted in the laboratory at 25 °C and 3000 lx using mated females, 2 to 7 days (average 4.8 days) old after emergence. These females had no oviposition experience because they were reared individually in plastic cups without host plants after mating. Prior to the bioassays, females were given 15% sucrose solution and then placed in a plastic chamber (33 × 42 × 24 cm) under 100 W incandescent light and allowed to fly freely inside for 1 h to activate their behavior. Each female was transferred to a test chamber containing one test leaf and allowed to oviposit freely for 3 h. To compensate for the effects of leaf tilt and light position on female choice, the leaf orientation was reversed 1.5 h after the start of the test. The number of eggs laid by each female on each treatment surface was recorded. Fifteen replicates were performed for each type of test leaf using different females.

### Feeding Bioassay

The effect of leaf wax on larval feeding preference was examined using two-choice tests, in which larvae were allowed to feed freely on one cabbage leaf with different wax traits in the left and right halves. Prior to the bioassays, each test leaf was placed between transparent glass plates and photographed using a TG-6 digital camera. The intact leaf area was determined using ImageJ 1.54d image analysis software. The left or right half of each test leaf was randomly subjected to one of the following combinations of surface treatments: (1) mechanical wax removal vs. no treatment (leaf wax intact), (2) wax removal, followed by spraying with solvent vs. wax removal, followed by spraying with an nC29 (5 mg) isopentane solution. After air-drying, each test leaf was placed vertically in a 5 mL glass vial containing water and placed at the center of a transparent cylindrical plastic case (20 cm height, 13 cm ID). To prevent the test leaves from drying or wilting, the glass vials were supplied with water every other day, and water-soaked cotton balls were placed inside the cases to maintain air moisture.

The bioassays were conducted in the laboratory at 25 °C, 3000 lx, and 16L:8D. Because early stage (first and early second instar) larvae feed on the leaves around their hatched eggs and rarely migrate (Tsuji et al. 2018), mid-stage (second to fourth instar) and final-stage (fifth instar) larvae were employed in the bioassays without sex distinction. Each larva was transferred onto the midvein of the test leaf and allowed to feed freely until the second instar larvae reached the fourth instar (average 6.1 days) and half a day for the fifth instar larvae. After the experiments, the test leaves were photographed using the method described above, and the leaf area (cm^2^) fed by the larvae on each treatment surface was measured using image analysis software. Fifteen replicates were performed for each type of test leaf using different larvae.

### Authentic Chemicals

nC29 (purity > 98%) and *n*-hentriacontane (purity > 95%) were purchased from Tokyo Chemical Industry (Tokyo, Japan). Triacontanal was synthesized via conventional pyridinium chlorochromate oxidation from authentic 1-triacontanol (purity > 85%) supplied by Tokyo Chemical Industry. After purification using silica gel column chromatography, the aldehyde product was obtained with a yield of > 26% and purity of > 90%.

### Data Analysis

Data analyses were conducted using R software version 4.3.1 (R Development Core Team 2023). In oviposition bioassays, to determine whether the type of leaf surface treatment influenced the likelihood of female oviposition, we employed a generalized linear mixed model (GLMM) with random effects of female identity. The modeling process was performed using the package ‘glmmML’ with a Poisson distribution. In feeding bioassays, significant differences in larval preference between the two different treatment surfaces were analyzed with the Wilcoxon signed-rank test using the package ‘exactRankTests.’

## Results

### Composition of Leaf Wax

**Fig. 1:**
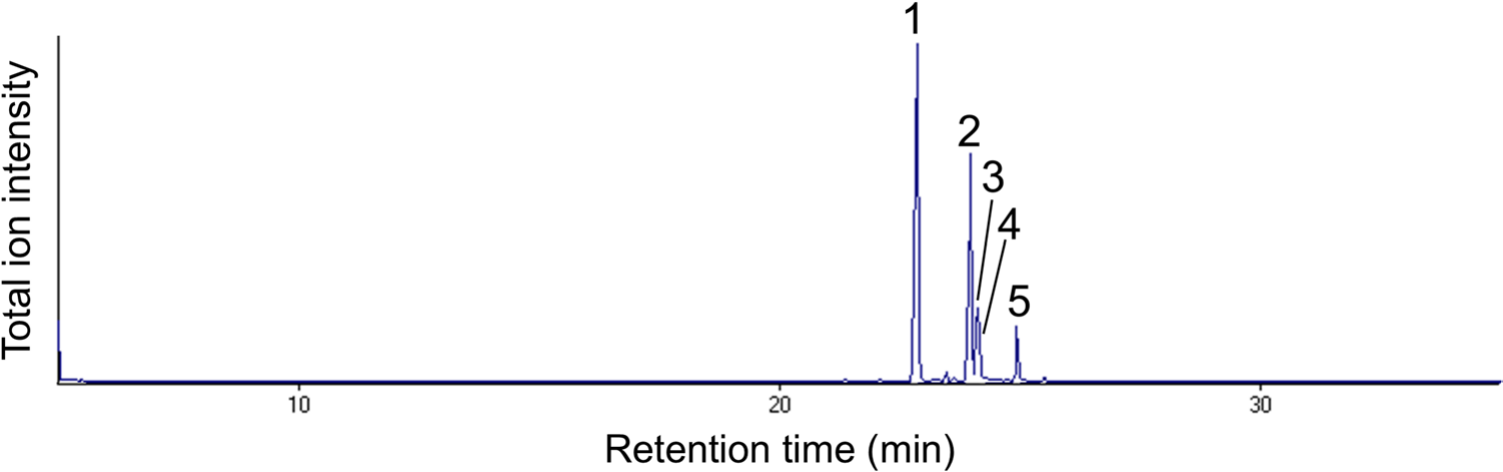
Representative total ion chromatogram of chloroform extract of intact cabbage cv. Kinkei 201 leaf. Chromatogram was run on a Supelco Equity-1 capillary column (15 m × 0.25 mm ID), programmed from 50 °C (initial 2 min hold) to 280 °C (final 10 min hold) at 10 °C/min. Peak numbers correspond to those in Table 1

Leaf epicuticular wax of cabbage cv. Kinkei 201 comprised 5 major components (Table 1; Fig. 1; see ion chromatogram of identified compounds in Online Resource 4). Among them, nC29 was the most predominant, and its amount per leaf area was 8.82 μg/cm^2^. Based on published data (Holloway and Brown 1977; Netting and Macey 1971), 15-nonacosanone and 15-nonacosanol, were tentatively assigned as the second and third major components, respectively. Triacontanal and *n*-hentriacontane were relatively minor components.

**Table 1:** Leaf surface wax composition from 7-week old seedlings of cabbage cv. Kinkei 201

No	Retention time (min)^a^	Retention index^b^	Compound	Amount^c^ (μg / cm^2^, mean ± SD, *N* = 10)
1	22.86	2900	*n*-Nonacosane	8.82 ± 1.96
2	23.96	3073	15-Nonacosanone^d^	4.55 ± 1.37
3	24.10	3095	15-Nonacosanol^d^	1.49 ± 0.49
4	24.13	3100	*n*-Hentriacontane	0.12 ± 0.09
5	24.93	3224	Triacontanal	0.66 ± 0.33

^a^On a Supelco Equity-1 capillary column (15 m × 0.25 mm ID), programmed from 50 °C (initial 2 min hold) to 280 °C (final 10 min) at a temperature increase of 10 °C/min

^b^Calculated from the retention times of authentic *n*-alkanes

^c^Calculated based on the peak intensity of *n*-nonacosane

^d^Tentative identification based on published data (Holloway and Brown 1977; Netting and Macey 1971)

### Modification of Leaf Surface Microstructures

**Fig. 2:**
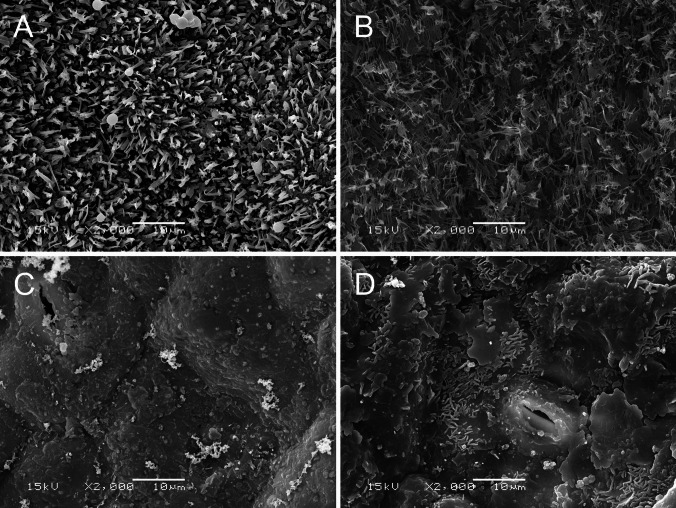
Scanning electron micrographs of cabbage cv. Kinkei 201 leaf surfaces used for bioassays. **a** Adaxial surface of an intact true leaf. **b** Abaxial surface of an intact true leaf. **c** Abaxial surface after rubbing with cotton balls. **d** Abaxial surface after rubbing with cotton balls and spraying with isopentane solution containing 5 mg authentic *n*-nonacosane

The glaucous surfaces of the cabbage leaves had crystalline structures of epicuticular wax, such as platelets partially containing tubules and rodlets with terminal dendrite branching (Fig. 2a and b). Wax removal treatment using cotton balls removed most of the crystals, exposing a smooth wax surface underneath (Fig. 2c). Spraying treatments with 5 mg nC29 produced wax crusts partially containing tubule crystals on the leaf surface (Fig. 2d).

### Effects of Wax Removal and Spraying Treatments on Female Oviposition

**Fig. 3:**
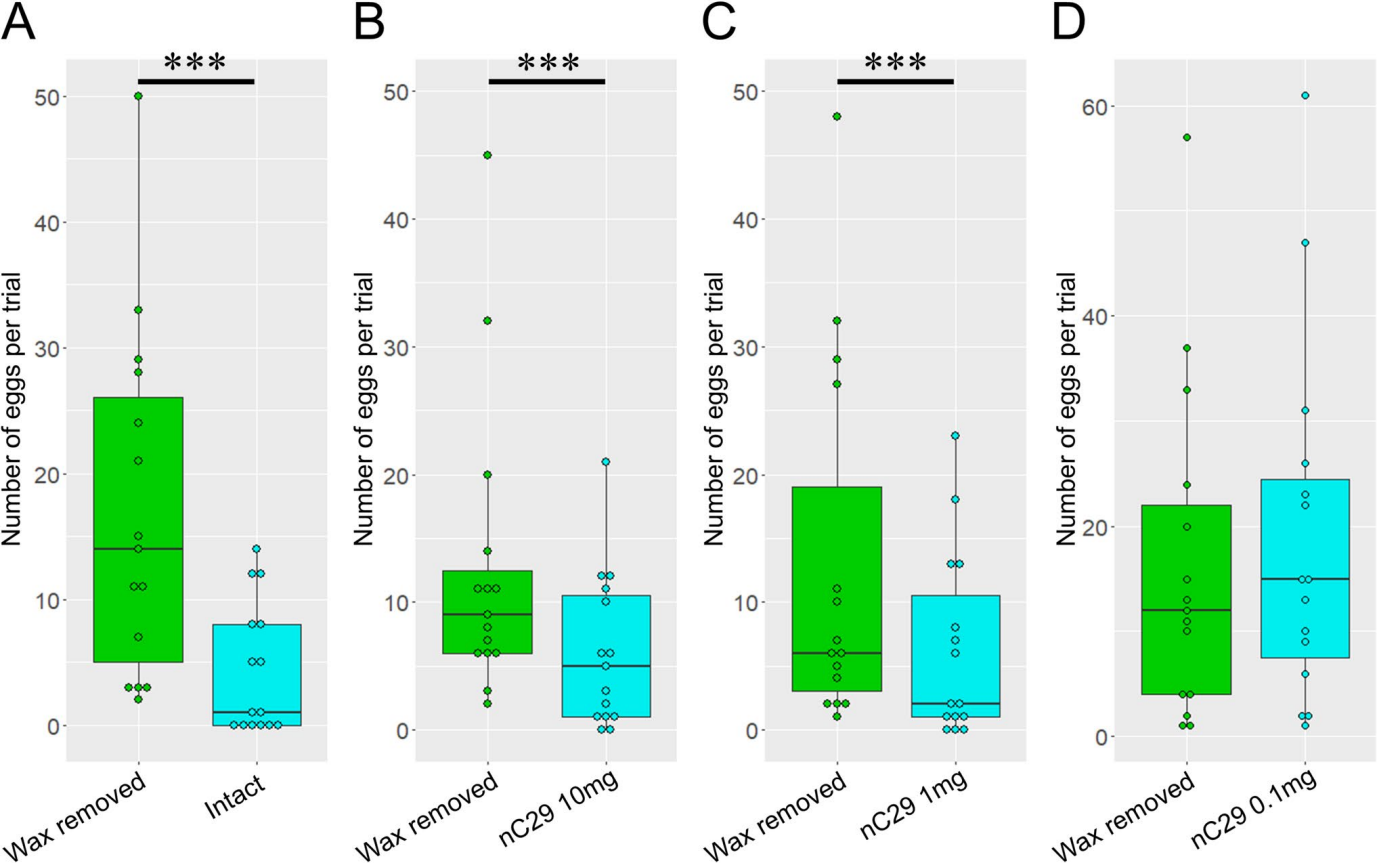
Oviposition preference of *Pieris rapae* female on one cabbage leaf with different treatments on the left and right halves from the midvein. **a** Wax-removed half vs intact half containing natural epicuticular wax. **b**-**d** Wax-removed half vs sprayed half with 10 mg, 1 mg, and 0.1 mg authentic *n*-nonacosane (nC29) after wax removal. Asterisks indicate that the presence of natural wax and nC29 on leaf surface had significant effects on the oviposition preference (GLMM: *** for *P* < 0.001)

Female oviposition preferences were negatively affected by the presence of innate epicuticular wax and the application of nC29 (Fig. 3). Mechanical wax removal from cabbage leaves using cotton balls significantly increased the number of eggs laid by females (Fig. 3a; GLMM, Z = −9.754, *P* < 0.001). Spraying 10 or 1 mg nC29 onto the wax-removed leaf surface significantly reduced the number of eggs laid by the females (Fig. 3b and c; GLMM, Z = −5.821 and −5.608, *P* < 0.001). However, spraying with 0.1 mg nC29 had no significant effect (Fig. 3d; GLMM, Z = 1.697, *P* = 0.090).

### Effects of Wax Removal and Spraying Treatments on Larval feeding

**Fig. 4:**
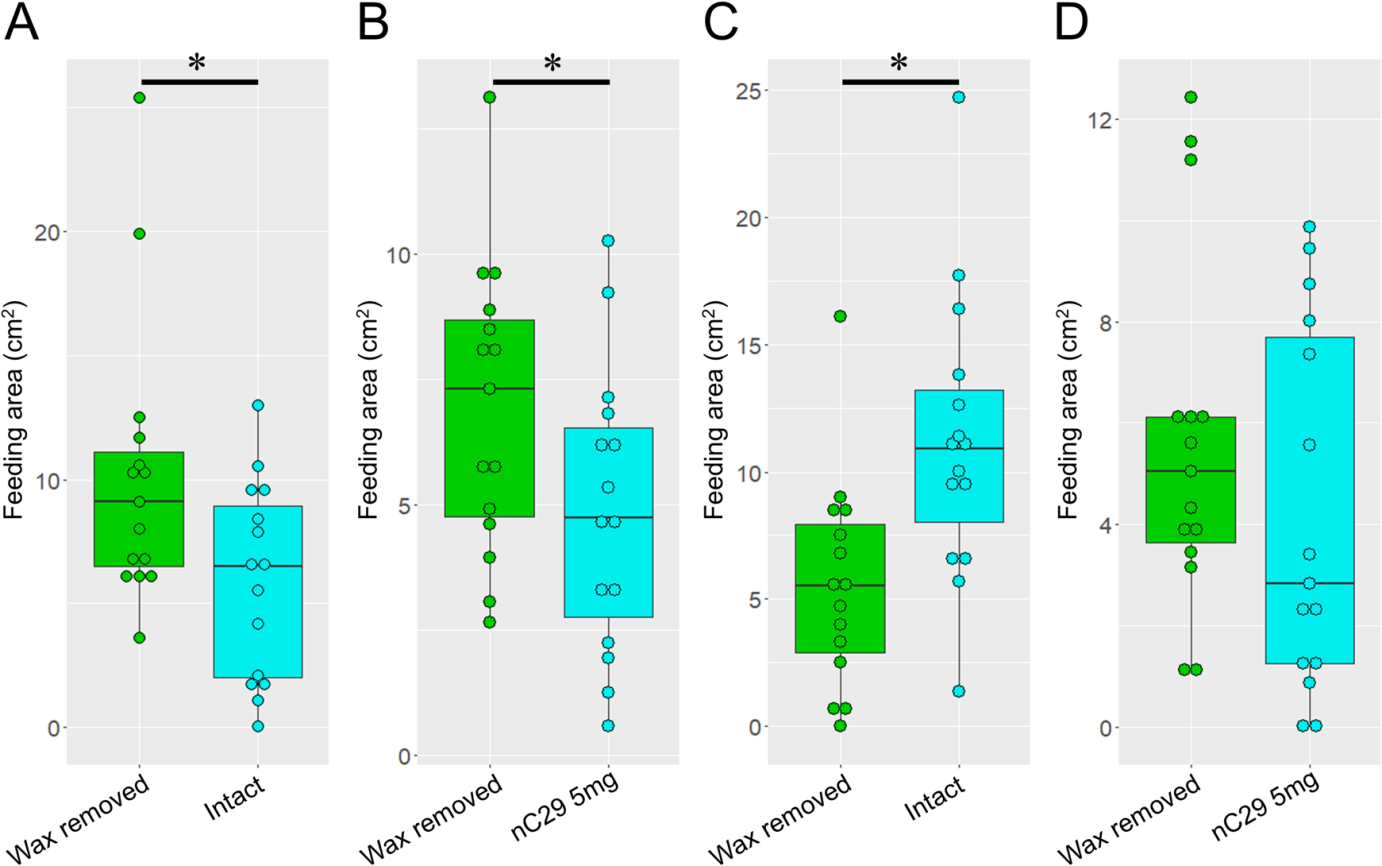
Feeding preference of *Pieris rapae* larvae on one cabbage leaf with different treatments on the left and right halves from the midvein. **a, c** Wax-removed half vs intact half containing natural epicuticular wax in trials for mid-stage (**a**) and final-stage (**c**) larvae. **b, d** Wax-removed half vs sprayed half with 5 mg authentic *n*-nonacosane (nC29) after wax removal in trials for mid-stage (**b**) and final-stage (**d**) larvae. Asterisks indicate that the presence of natural wax and nC29 on leaf surface had significant effects on the feeding preference (Wilcoxon signed-rank test: * for *P* < 0.05)

Larval feeding preferences were affected by different cabbage leaf surface treatments (Fig. 4). Mid-stage larvae significantly preferred to feed on wax-removed leaves than on intact leaves with innate epicuticular wax and treated leaves with wax-removed and sprayed with 5 mg nC29 (Fig. 4a and b; Wilcoxon signed-rank test, V = 18 and 20, *P* < 0.05). In contrast, final-stage larvae significantly preferred intact leaves to wax-removed leaves (Fig. 4c; Wilcoxon signed-rank test, V = 105, *P* < 0.05), but difference in choice between wax-removed leaves and those treated with 5 mg nC29 was not significant (Fig. 4d; Wilcoxon signed-rank test, V = 34, *P* = 0.151).

## Discussion

This study revealed that the leaf epicuticular wax of cabbage cv. Kinkei 201 and its predominant component nC29 suppressed female oviposition and mid-stage larval feeding of *P*. *rapae* under laboratory conditions. Female adults and larvae tend to avoid leaf surfaces with complex microstructures, indicating that cabbage leaf wax has physical influences on their host selection and preference. In Lepidoptera, several moth species including *Diaphania indica* (Crambidae; Debnath et al. 2021), *Spilosoma obliqua* (Arctiidae; Mobarak et al. 2020; Roy 2021), *Ostrinia latipennis* (Crambidae; Li and Ishikawa 2006), *Helicoverpa armigera* (Noctuidae: Roy 2021), and *Spodoptera litura* (Noctuidae: Roy 2021) use leaf wax and its components of host plants for short-range orientation and oviposition. On the other hand, a few moth species such as *Spodoptera frugiperda* (Noctuidae: Rojas et al. 2003) and *P*. *xylostella* (Zhu et al. 2022) have reported a low preference for leaves with high wax contents.

Cabbage cv. Kinkei 201 had a leaf wax profile similar to those of other common cultivars such as CW1 (Cao et al. 2021; Laila et al. 2017). The predominant component was nC29, whereas the other major components were 15-nonacosanone, 15-nonacosanol, triacontanal, and *n*-hentriacontane. The main structures of these wax components are formed by carbon-elongation reaction of C_16_ and C_18_ acyl-coenzyme A, and functional groups are formed by various reactions, such as oxidation and decarbonation (Lewandowska et al. 2020). These molecules are biosynthesized in epidermal cells and transported through the cell wall to the cuticle (Samuels et al. 2008). On the cuticular surface, they form a characteristic crystalline structure called ‘polymorphism’, which varies depending on growth conditions and wax composition (Barthlott et al. 1998; Koch and Ensikat 2008).

Adult females and mid-stage larvae of *P*. *rapae* significantly preferred the wax-removed leaves to the intact leaves and nC29-treated leaves after wax removal. The low preference of larvae for waxy leaves was consistent with that of adult females, suggesting that females avoid laying eggs on the leaves with high crystalline wax contents, which are unsuitable as larval food. These results indicate that cabbage leaf epicuticular wax and nC29 play potential roles in the defense against the brassicaceous specialist *P*. *rapae*, although their defensive effects are small and inconspicuous under natural conditions. Our findings are not consistent with the results of previous studies showing that glossy varieties of *Brassica* crops with low wax contents exhibit higher resistance to *P*. *rapae*. (Shelton et al. 1988; Stoner 1990, 1992). One possible reason for this discrepancy is that previous studies examined larval populations and plant damage under field conditions in which larval and plant growth are affected by various biotic and abiotic factors, including other herbivores, predators, microbes, temperature, precipitation, and drought. The host suitability of glossy cabbage varieties and behavioral responsiveness of *P*. *rapae* to them should be reinvestigated under controlled laboratory conditions.

Unlike mid-stage larvae, final-stage larvae significantly preferred the intact leaves containing natural wax to the wax-removed leaves. This result suggests that susceptibility and resistance to leaf wax change during larval development. Lepidopteran larvae exhibit higher detoxification capacity against phytochemicals and pesticides in later stages (Jeschke et al. 2017; Yu and Hsu 1993). In addition, it may be due to their learning to associate leaf wax with food because they were reared on cabbages with a wax profile similar to that of Kinkei 201 until just before the bioassays. Phytophagous insects are known to acquire feeding preferences for the hosts that they used in early stages (Bernays and Weiss 1996; Jermy et al. 1968).

Several previous studies reported that brassicaceous plants have glucosinolates on the leaf surface, which stimulate female oviposition and larval feeding of Brassicaceae-feeding insects (Badenes-Pérez et al. 2011; Griffiths et al. 2001). Thus, the mechanical wax removal in this study might affect (decrease) the content of leaf-surface glucosinolates, causing differences in oviposition and feeding preferences between treated and intact surfaces in *P*. *rapae*. On the other hand, another study revealed that glucosinolates are rarely present in the epidermis, suggesting that adult females of *Pieris* may sense them present in the inside layer of cuticular proper and in the cuticular layer, or through stomata (Städler and Reifenrath 2009). To more accurately evaluate the effect of leaf surface wax on host selection in *P*. *rapae*, future studies should be conducted using plastic leaf models and artificial diets, which contain glucosinolates at concentrations sufficient to induce female oviposition and larval feeding.

Conflicting results have been reported regarding the effects of leaf wax on the host selection of *P*. *xylostella*. The female adults and larvae of *P*. *xylostella* prefer brassicaceous crops with low wax content (Eigenbrode et al. 1991; Justus et al. 2000; Zhu et al. 2022). However, female adults do not distinguish between glossy and waxy varieties of *Brassica rapa* for oviposition, but first-instar larvae strongly prefer waxy varieties for feeding (Ulmer et al. 2002). *n*-Alkane constituents of cabbage leaf wax synergistically enhance female oviposition responses to cabbage homogenate and an authentic glucosinolate, sinigrin (Spencer 1996; Spencer et al. 1999). Based on these findings, leaf epicuticular wax may have both positive and negative effects on lepidopteran host selection depending on plant species and variety, insect growth stage, and interactions with other chemicals.

The mechanisms by which cabbage leaf wax and nC29 affect the host selection of *P*. *rapae* larvae and adults will be the subject of future studies. One probability is that the wax components physically inhibit female oviposition and mid-stage larval feeding by forming microcrystal structures on the leaf surface. The complexity of the wax bloom microstructure is strongly related to plant resistance to certain herbivores by impeding their attachment to the leaf surface (Eigenbrode and Espelie 1995). This effect is particularly pronounced in neonatal larvae because of their small body size (Eigenbrode et al. 1991). Another possibility is that the wax components serve as allelochemicals (deterrents) in larval feeding and female oviposition. There is a limited knowledge on chemosensory responses of herbivorous insects to long-chain *n*-alkanes in host selection. The plant bug *Lygus pratensis* (Hemiptera: Miridae) exhibits antennal olfactory responses to *n*-heptacosane and *n*-octacosane standards despite their low volatility (Feng et al. 2022). The chrysomelid leaf beetle *Galerucella grisescens* (Coleoptera: Chrysomelidae) responds to the leaf wax extract of its host plant *Rumex obtusifolius* (Polygonaceae) during tarsal gustation (Yosano et al. 2020).

The composition and crystalline structure of leaf epicuticular wax in *Brassica* spp. differ greatly depending on the environment, leaf age, and leaf position (Baker 1974; Laila et al. 2017; Shepherd et al. 1995). Thus, the effects of leaf epicuticular wax on host selection by phytophagous insects are likely to vary spatially and temporally, even within the same plant species. This is consistent with previous studies showing that the host preferences of *P*. *rapae* and *P*. *xylostella* for waxy varieties of *Brassica* crops vary depending on the test conditions. Further studies are needed to determine changes in leaf wax profile with plant growing conditions and development and their effects on host preference of *P*. *rapae*.

In conclusion, we found that nC29 was the most predominant leaf epicuticular wax present in cabbage cv. Kinkei 201. Female oviposition on cabbage leaf surfaces was suppressed by innate epicuticular wax and nC29 application. Moreover, mid-stage larvae preferred to feed on wax-removed leaves than on intact leaves with innate epicuticular wax and nC29-sprayed leaves after wax removal. Our findings reveal that cabbages with higher wax contents and well-developed wax blooms may be more resistant to *P*. *rapae*. Thus, to reduce and control herbivorous damages caused by *P*. *rapae*, acquiring these leaf traits in cabbages through improved breeding and cultivation methods is important. The present results will provide the basis for future studies to determine the mechanisms by which cabbage leaf wax and nC29 affect the host selection by *P*. *rapae*.

## Supplementary Information

Below is the link to the electronic supplementary material.

ESM 1 (DOCX 51.3 KB)

ESM 2 (DOCX 51.3 KB)

ESM 3 (DOCX 29.1 KB)

ESM 4 (DOCX 139 KB)

## Data Availability

The data supporting the findings of this study are available from the corresponding author upon reasonable request.
