# Supplementary material for: Cabbage Leaf Epicuticular Wax Deters Female Oviposition and Larval Feeding of Pieris rapae
Source: J Chem Ecol. 2025 Mar 25;51(2):45. doi: 10.1007/s10886-025-01597-z (PMC11937181; doi:10.1007/s10886-025-01597-z)
Supplement: Supplementary file 1 — (DOCX 51.3 KB) [file 10886_2025_1597_MOESM1_ESM.docx]

**Online Resource 1**

Cabbage leaf epicuticular wax deters female oviposition and larval feeding of *Pieris* *rapae*

Itsuki Ueno^1^, Taisei Kanedawara^2^, Kodai Inoue^1^, Sotaro Watanabe^2^, Hisashi Ômura^1,3^

^1^ Graduate School of Integrated Sciences for Life, Hiroshima University

^2^ School of Applied Biological Science, Hiroshima University

^3^ Seto Inland Sea Carbon-neutral Research Center, Hiroshima University

**Calibration curve of authentic *n*-nonacosane**

Of the five major components identified from leaf wax extracts of cabbage cv. Kinkei 201, *n*-nonacosane (nC29) was the most predominant and was commercially available. Accordingly, this compound was used as an external standard for quantification of epicuticular wax components. Test solutions were prepared by dissolving 10 mg and 1 mg authentic nC29 in 10 mL chloroform, respectively. These solutions were subjected to gas chromatography-electron impact mass spectrometry (GC-EIMS), and a calibration curve was constructed based on the linear relationship between sample amount and peak area in total ion chromatogram (TIC). GC-EIMS was carried out an EI voltage of 70 eV using a QP5000 mass spectrometer (Shimadzu, Kyoto, Japan) and GC-17A gas chromatograph (Shimadzu, Kyoto, Japan) equipped with a Supelco Equity-1 capillary column (15 m × 0.25 mm ID, 0.25 μm film thickness: Bellefonte, PA, USA). The splitless injection of 1 μL sample (containing 1 μg or 0.1 μg of nC29) was operated with an injector temperature of 280 °C and a split opening 30 s after injection. The oven temperature was programmed from 50 °C (initial 2 min hold) to 280 °C (final 10 min hold) at 10 °C/min.

**Result**

Table 1 Raw data of the analyses

| nC29 amount (µg) | Peak area in TIC |
| --- | --- |
| 1 | 289206770 |
| 0.1 | 27594820 |


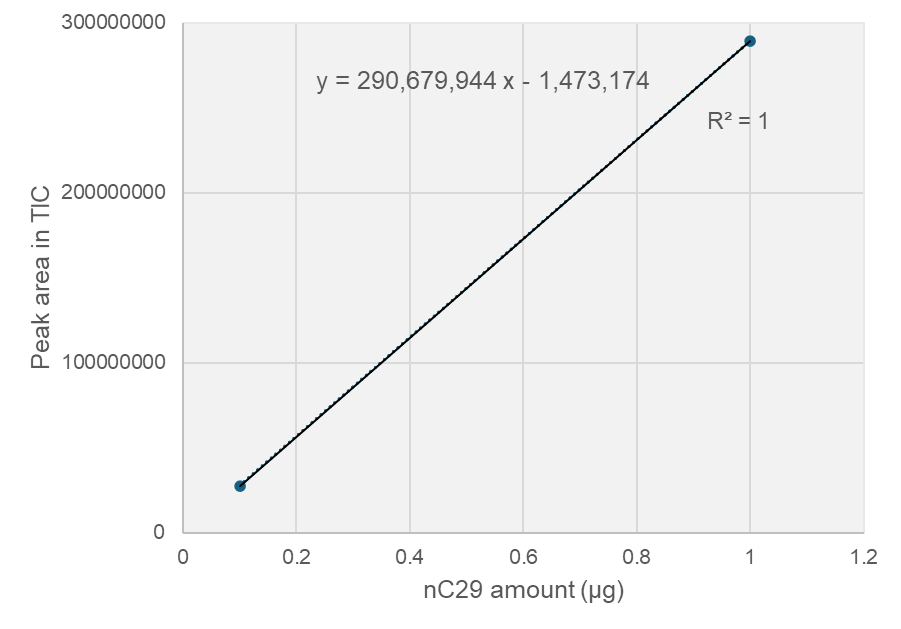


**Fig. 1** Calibration curve of authentic *n*-nonacosane (nC29) in GC-EIMS

The following linear regression equation (y: peak area, x: nC29 amount) was obtained from the calibration curve for nC29.

$$y=290679940 x-1473174$$
