# Supplementary material for: Cabbage Leaf Epicuticular Wax Deters Female Oviposition and Larval Feeding of Pieris rapae
Source: J Chem Ecol. 2025 Mar 25;51(2):45. doi: 10.1007/s10886-025-01597-z (PMC11937181; doi:10.1007/s10886-025-01597-z)
Supplement: Supplementary file 2 — (DOCX 51.3 KB) [file 10886_2025_1597_MOESM2_ESM.docx]

**Online Resource 2**

Cabbage leaf epicuticular wax deters female oviposition and larval feeding of *Pieris* *rapae*

Itsuki Ueno^1^, Taisei Kanedawara^2^, Kodai Inoue^1^, Sotaro Watanabe^2^, Hisashi Ômura^1,3^

^1^ Graduate School of Integrated Sciences for Life, Hiroshima University

^2^ School of Applied Biological Science, Hiroshima University

^3^ Seto Inland Sea Carbon-neutral Research Center, Hiroshima University

**Quantification of wax remaining on cabbage leaves after mechanical wax removal**

True leaves were detached at the base of the petiole from 5-week-old seedlings of cabbage cv. Kinkei 201. From either the right or left half from the midvein of a single leaf, epicuticular wax was mechanically removed by rubbing both the adaxial and abaxial sides of the leaf with clean cotton balls. On the other hand, on the opposite half of the leaf, the innate epicuticular wax remained intact. Each half of a single leaf was soaked in 10 mL dichloromethane in a petri dish for 30 s to extract the intact wax and the wax remaining after the mechanical wax removal, respectively. After extraction, each test leaf was air-dried to volatilize the solvent, placed between transparent glass plates, and photographed with a TG-6 digital camera (Olympus, Tokyo, Japan) to determine the leaf area of each half using ImageJ 1.54d software. This sampling, in which two extracts were obtained from one cabbage leaf, was repeated three times using cabbage leaves from different seedlings.

The extracts obtained were immediately subjected to gas chromatography-electron impact mass spectrometry (GC-EIMS). Prior to analyses, an 1 mL portion of each sample was concentrated to 200 μL under a gentle nitrogen stream at 60 °C. GC-EIMS was carried out an EI voltage of 70 eV using a QP5000 mass spectrometer (Shimadzu, Kyoto, Japan) and GC-17A gas chromatograph (Shimadzu, Kyoto, Japan) equipped with a Supelco Equity-1 capillary column (15 m × 0.25 mm ID, 0.25 μm film thickness: Bellefonte, PA, USA). The splitless injection of 1 μL sample concentrated was operated with an injector temperature of 280 °C and a split opening 30 s after injection. The oven temperature was programmed from 50 °C (initial 2 min hold) to 280 °C (final 10 min hold) at 10 °C/min. The amount of *n*-nonacosane, the most predominant component in extracts, was quantified with a calibration curve of its authentic compound, and then the content per area was calculated for each half of the leaf.

**Result**

The mechanical removal treatments with cotton balls obviously changed the cabbage surface from glaucous to glossy. The average content of *n*-nonacosane per leaf area was calculated 19.4 μg/cm^2^ for intact half and 7.0 μg/cm^2^ for wax-removed half, respectively (Table 1; Fig. 1). This result shows that the mechanical removal treatments reduced the content of *n*-nonacosane to average of 37% its original content in the untreated leaves

**Table 1** Comparison of wax contents between intact and wax-removed half leaves.

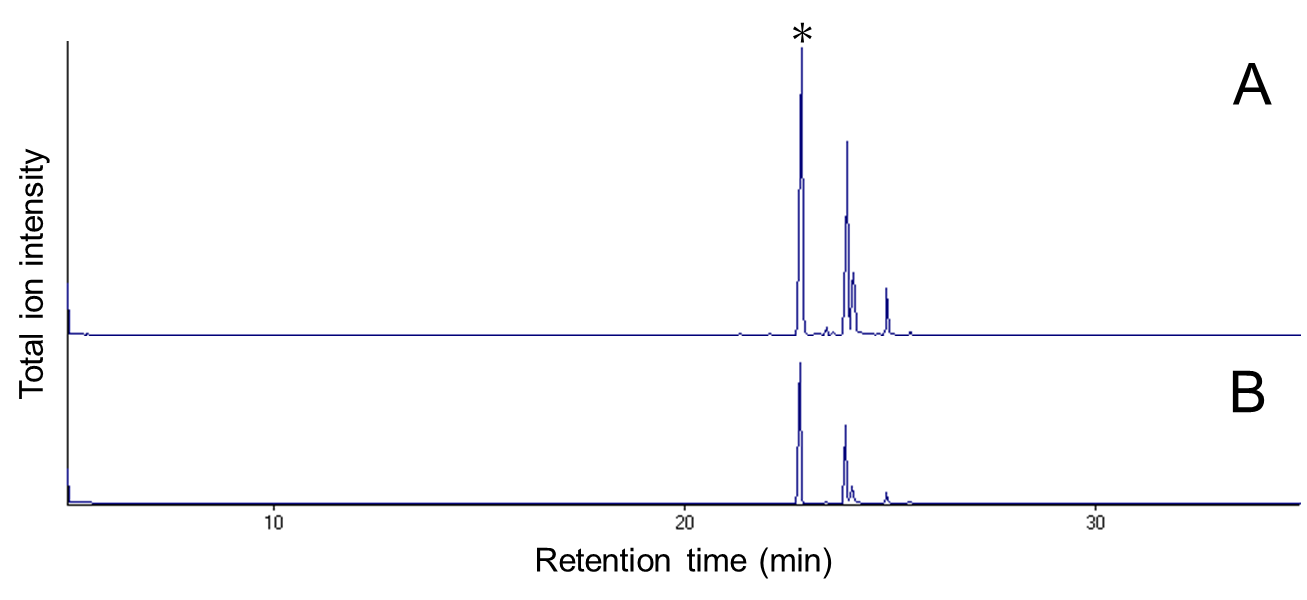


**Fig. 1** Typical total ion chromatograms of chloroform extracts from intact half (**a**) and mechanically-wax-removed half (**b**) leaves of cabbage cv. Kinkei 201. The vertical axes of the two traces are at the same scale. Peaks with an asterisk denote *n*-nonacosane.
