# Supplementary material for: Cabbage Leaf Epicuticular Wax Deters Female Oviposition and Larval Feeding of Pieris rapae
Source: J Chem Ecol. 2025 Mar 25;51(2):45. doi: 10.1007/s10886-025-01597-z (PMC11937181; doi:10.1007/s10886-025-01597-z)
Supplement: Supplementary file 3 — (DOCX 29.1 KB) [file 10886_2025_1597_MOESM3_ESM.docx]

**Online Resource 3**

Cabbage leaf epicuticular wax deters female oviposition and larval feeding of *Pieris* *rapae*

Itsuki Ueno^1^, Taisei Kanedawara^2^, Kodai Inoue^1^, Sotaro Watanabe^2^, Hisashi Ômura^1,3^

^1^ Graduate School of Integrated Sciences for Life, Hiroshima University

^2^ School of Applied Biological Science, Hiroshima University

^3^ Seto Inland Sea Carbon-neutral Research Center, Hiroshima University

**Quantification of wax on cabbage leaves sprayed with authentic compound**

True leaves were detached at the base of the petiole from 5-week-old seedlings of cabbage cv. Kinkei 201. From both abaxial and adaxial sides of a single leaf, epicuticular wax was mechanically removed by rubbing the surface with cotton balls. Half of the wax-removed leaf from the midvein was covered with aluminum foil. Then the uncovered opposite half was sprayed on both abaxial and adaxial sides with authentic *n*-nonacosane (nC29: 0.1, 1, and 10 mg) dissolved in 7 mL isopentane using a hobby airbrush. After removing the aluminum foil cover, each half of a single leaf was soaked separately in 40 mL dichloromethane in glass vial for 30 s to extract the surface wax. After extraction, t each test leaf was air-dried to volatilize the solvent, placed between transparent glass plates, and photographed with a TG-6 digital camera to determine the leaf area of each half using ImageJ 1.54d software. This sampling, in which two extracts were obtained from one cabbage leaf, was repeated three times for one dose of nC29, using different leaves.

The extracts obtained were immediately subjected to gas chromatography-electron impact mass spectrometry (GC-EIMS). Prior to analyses, an 1 mL portion of each sample was concentrated to 200 μL under a gentle nitrogen stream at 60 °C. GC-EIMS was carried out an EI voltage of 70 eV using a QP5000 mass spectrometer (Shimadzu, Kyoto, Japan) and GC-17A gas chromatograph (Shimadzu, Kyoto, Japan) equipped with a Supelco Equity-1 capillary column (15 m × 0.25 mm ID, 0.25 μm film thickness: Bellefonte, PA, USA). The splitless injection of 1 μL sample concentrated was operated with an injector temperature of 280 °C and a split opening 30 s after injection. The oven temperature was programmed from 50 °C (initial 2 min hold) to 280 °C (final 10 min hold) at 10 °C/min. The amount of nC29 was quantified with a calibration curve of its authentic compound, and then the content per area was calculated for each half of the leaf.

**Result**

The average content of nC29 per unit area was calculated to be 8.4 μg/cm^2^ for half leaves with wax mechanically removed (Table 1). When 0.1, 1, and 10 mg of authentic nC29 was sprayed on both sides of wax-removed half leaves, its average content was calculated to be 6.8, 55.8, and 360.1 μg/cm^2^, respectively (Table 1). Intact leaves contain an average of 19.4 μg/cm^2^ of nC29 (Online Resource 1). The nC29 content per unit area depends on the total surface area of treated leaf, but spraying 0.1 mg of the authentic compound to a wax-removed leaf does little to increase its content. On the other hand, if more than 1 mg of its authentic compound is applied, the content will be greater than that found in intact leaves.

**Table 1** Comparison of *n*-nonacosane content in half leaves with wax removed and those with wax removed and sprayed with 0.1, 1, and 10 mg its authentic compound
