# Supplementary material for: Cabbage Leaf Epicuticular Wax Deters Female Oviposition and Larval Feeding of Pieris rapae
Source: J Chem Ecol. 2025 Mar 25;51(2):45. doi: 10.1007/s10886-025-01597-z (PMC11937181; doi:10.1007/s10886-025-01597-z)
Supplement: Supplementary file 4 — (DOCX 139 KB) [file 10886_2025_1597_MOESM4_ESM.docx]

**Online Resource 4**


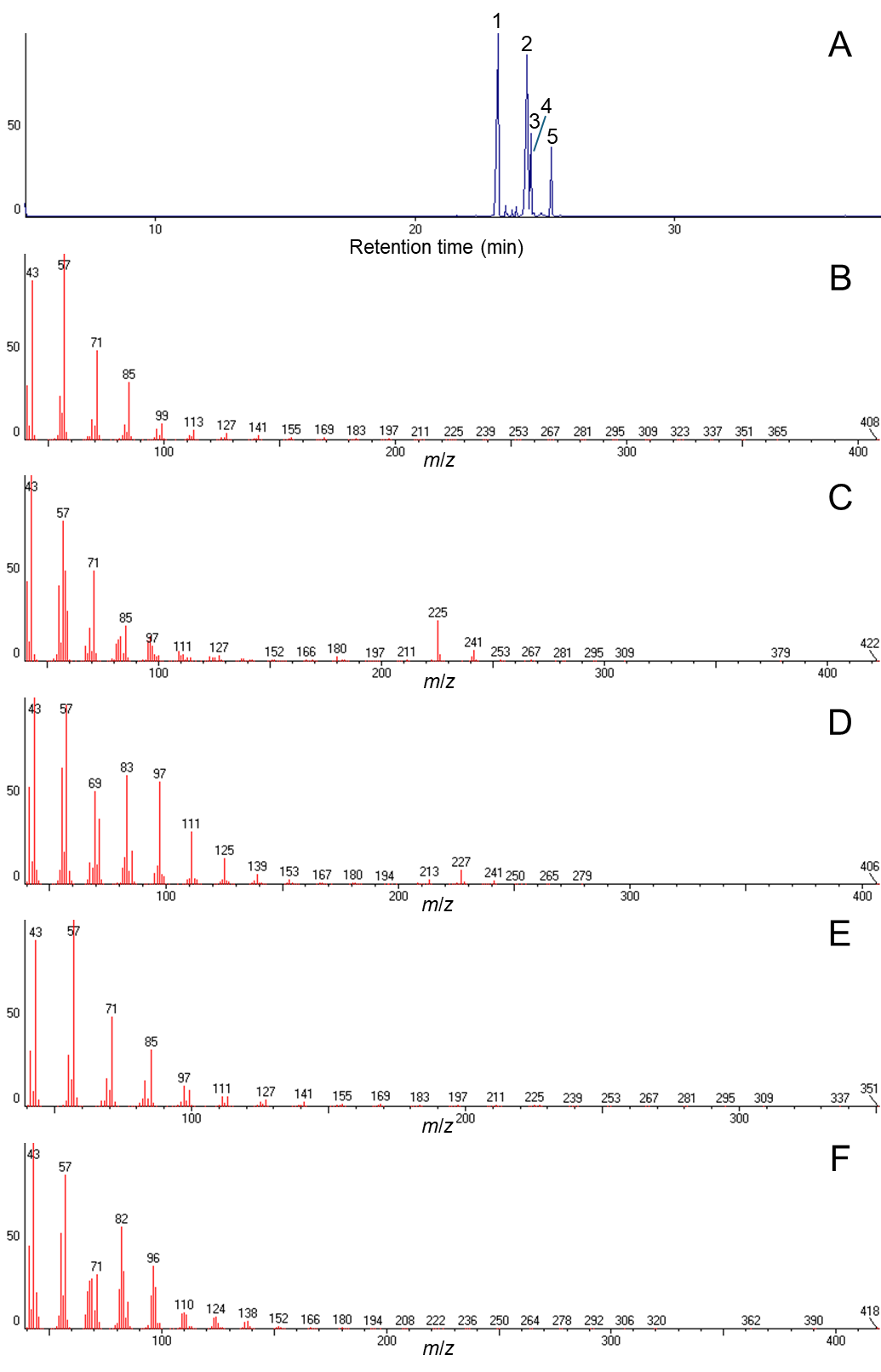


**Fig.S1** Total ion chromatogram obtained from chloroform extract of leaf epicuticular wax of cabbage cv. Kinkei 201 (**a**) and mass chromatograms of major peaks 1–5 (**b**–**f**). Peaks 1–5 correspond to *n*-nonacosane, 15-nonacosanone, 15-nonacosanol, *n*-hentriacontane, and triacontanal, respectively.
